# Trying to Understand the Complicated Taxonomy in *Amaranthus* (Amaranthaceae): Insights on Seeds Micromorphology

**DOI:** 10.3390/plants12050987

**Published:** 2023-02-21

**Authors:** Duilio Iamonico, Amara Noor Hussain, Arya Sindhu, Venugopalan nair Saradamma Anil Kumar, Shabnum Shaheen, Mamoona Munir, Paola Fortini

**Affiliations:** 1 Department of Environmental Biology, University of Rome Sapienza, Piazzale Aldo Moro 5, 00185 Rome, Italy; 2 Department of Bioscience and Territory, University of Molise, Fonte Lappone, 86090 Pesche, Italy; 3 Department of Botany, University College, Thiruvananthapuram 695034, Kerala, India; 4 Department of Botany, Government College, Kasaragod 671123, Kerala, India; 5 Department of Botany, Lahore College for Women University, Jail Road, Lahore 54000, Pakistan; 6 Department of Botany Rawalpindi, Women University, Satellite Town Rawalpindi, Islamabad 46300, Pakistan

**Keywords:** *Amaranthus*, classification, coat ornamentation, SEM, species, statistical analysis, taxa

## Abstract

*Amaranthus* is a genus taxonomically complex because of its high morphological variability, which led to nomenclatural disorders, misapplication of names, and misidentifications. Floristic and taxonomic studies on this genus are still incomplete, and many questions remain open. Seed micromorphology has been shown to play an important role in the taxonomy of plants. Regarding Amaranthaceae and *Amaranthus*, investigations are rare, and they refer to one or a few species. With the primary aim to test if seed features are helpful in the taxonomy of *Amaranthus*, we here present a detailed SEM study on seed micromorphology in 25 *Amaranthus* taxa using morphometric methods. Seeds were collected from field surveys and herbarium specimens; 14 seed coat features (7 qualitative and 7 quantitative) were measured on 111 samples (up to 5 seeds per sample). The results obtained revealed that seeds micromorphology provides interesting new taxonomic data concerning some taxa (species and below ranks). In fact, we were able to distinguish a few seed types, including one or more taxa, i.e., *blitum*-type, *crassipes*-type, *deflexus*-type, *tuberculatus*-type, and *viridis*-type. On the other hand, seed features are not useful for other species, for example, those included in the *deflexus*-type (*A. deflexus*, *A. vulgatissimus*, *A. cacciatoi*, *A. spinosus*, *A. dubius*, and *A. stadleyanus*). A diagnostic key of the studied taxa is proposed. Subgenera cannot be distinguished using seed features, thus confirming the published molecular data. All these facts reveal, once again, the taxonomic complexity of the genus *Amaranthus* since, e.g., just a few seed types can be defined.

## 1. Introduction

*Amaranthus* L. (Amaranthaceae Juss., Caryophyllales Perleb.) is a genus comprising 70–80 species, of which approximately half are native to the Americas [1,2,3]; few taxa occur naturally in the other continents, (e.g., [1,4]). Many *Amaranthus* species can spread out of their native distribution areas and sustain self-replacing populations, negatively impacting both the agricultural systems and natural vegetation (e.g., [3,5,6]).

The genus *Amaranthus* is taxonomically complicate, being characterized by a high phenotypic variability, which has resulted in nomenclatural confusion and misapplication of names (e.g., [5,7,8,9,10,11,12,13]).

Mosyakin & Robertson [14] proposed a classification of *Amaranthus* recognizing three subgenera, i.e., subgenus *Acnida* (L.) Aellen ex K.R. Robertson (dioecious species), subgenus *Albersia* (Kunth) Gren. & Godr. (monoecious species with usually two–three tepals and synflorescence usually arranged in axillary glomerules), and subgenus *Amaranthus* (monoecious species with mostly five tepals and synflorescence arranged in elongated spike- or panicle-like structures). Furthermore, they proposed three sections for subgen. *Acnida* [sect. *Acnida* (L.) Mosyakin & K.R. Robertson, sect. *Saueranthus* Mosyakin & K.R. Robertson, and sect. *Acanthochiton* (Torr.) Mosyakin & K.R. Robertson], four sections for subgen. *Albersia* [sect. *Blitopsis* Dumort., sect. *Pentamorion* (G.Beck) Mosyakin & K.R.Robertson, sect. *Goerziella* (Urban) Mosyakin & K.R.Robertson, and sect. *Pyxidium* Moq.], and three sections for subgen. *Amaranthus* (sect. *Amaranthus*, sect. *Dubia* Mosyakin & K.R.Robertson, and sect. *Centrusa* Griseb). A recent molecular study by Waselkov et al. [15] highlighted that the classification by Mosyakin & Robertson [14] is not natural, not matching the clades as identified in the phylogenetic trees.

In addition to the taxonomic issues, the nomenclature of *Amaranthus* is also highly complicated, especially for the misinterpretations of the names, which cause, e.g., the use of different names for the same taxon (for example, *A. chlorostachys* Willd. = *A. patulus* Bertol. = *A. hybridus* L. [8]), or the use of a name for a wrong taxon (for example *A. gracilis sensu auct. non* Desfontaines, which is supposed to be referred to as *A. viridis* L. [9]), or the occurrence of ambiguous names (for example *A. gangeticus* L. [16]).

Seed micromorphology has been shown to often play an essential role in the taxonomy of numerous plant groups, including Caryophyllales Perleb., e.g., Aizoaceae Martinov on Sesuvioideae Lindl [17], Chenopodiaceae Vent. on *Chenopodium* L. and related genera [18], Cactaceae Juss. on *Melocactus* Link & Otto [19], on *Stenocereus* Riccob [20], Caryophyllaceae Juss. on *Arenaria* L. [21], on *Gypsophila* L. [22], on *Moehringia* L. [23], on *Silene* L. [24], Polygonaceae Juss. on *Polygonum* L. [25]. Concerning Amaranthaceae Juss. (79 genera, according to [2]), studies on the micromorphology of seed coats are rare, referring to one to a few species, e.g., *Aerva javanica* (Brum.f.) Juss. ex Schultz [26] or *Allmania nodiflora* (L.) R.Br. & Wight and *A. multiflora* S. Arya, V.S.A. Kumar, V. Suresh & Iamonico [27]. In regard to the genus *Amaranthus*, a few papers were published referring to one (e.g., *A. hybridus* L. [28], *A. tuberculatus* (Moq.) J.D. Sauer [29]) or a few taxa [30,31,32].

As part of the ongoing studies on the taxonomy and nomenclature of the genus *Amaranthus* (by DI, see, e.g., [8,9,10,11,12,16,33,34,35,36,37,38,39]), here we present a detailed study on seed micromorphology in *Amaranthus*. The primary aim of the study is to test if seed features are helpful in the taxonomy of the genus at both subgenus and species ranks, also considering the molecular study by Waselkov et al. [15]. The number of taxa we considered is 25, much greater than those included in the previously published papers (e.g., [26,27,28,29,30,31,32]).

## 2. Materials and Methods

### 2.1. Plant Material

The research is based on our field investigations carried out in Italy from 2019 to 2022. Plants collected are deposited at the Herbaria IS and RO. Further seed material was taken from the following Herbaria: BA, CANB, HAL, LPAG, M, PERTH, PNUH, RO, and SI (acronyms follow Thiers [40]) (see Appendix A for the list of specimens).

**Figure 1:**
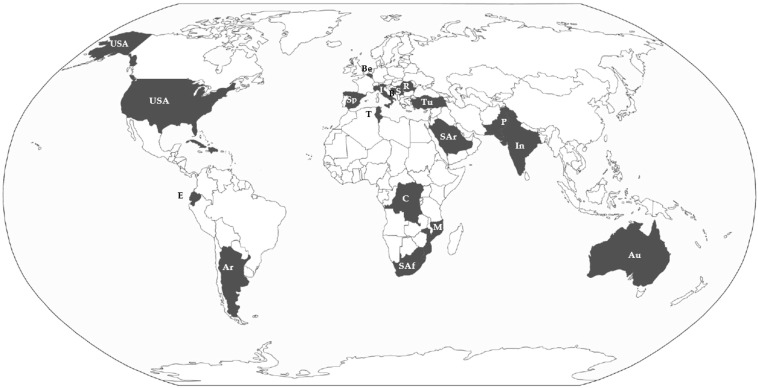
Map of the countries referring to the studied samples. Abbreviations: Ar = Argentina, Au = Australia, B = Bosnia and Herzegovina, Be = Belgium, C = Congo Democratic Republic, E = Equador, In = India, M = Mozambique, P = Pakistan, R = Romania, S = Serbia, Sar = Saudi Arabia, Sp = Spain, SAf = Republic of South Africa, Tu = Turkey, USA = United States of America.

**Figure 2:**
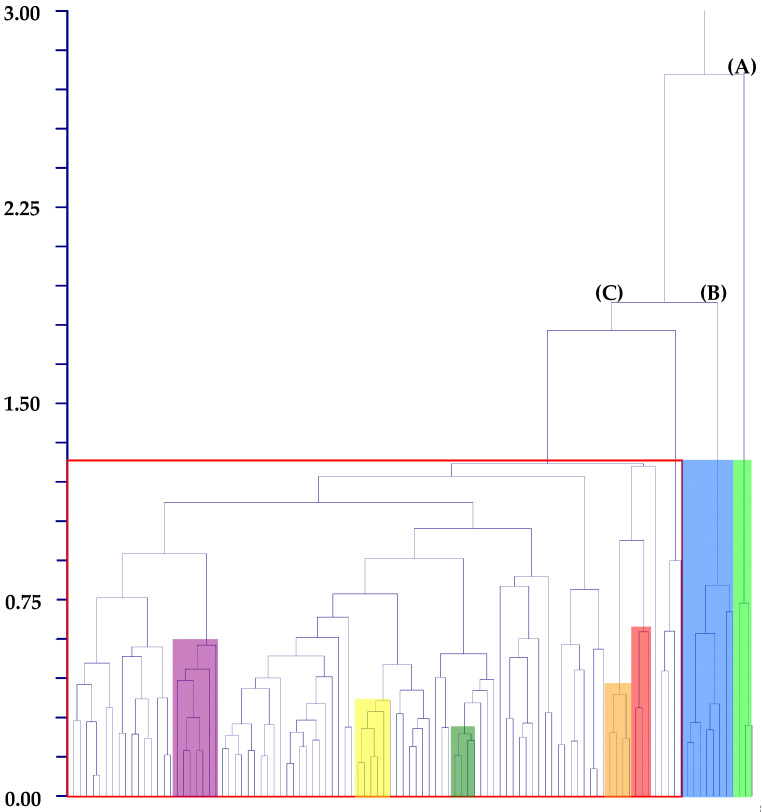
Dendrogram graph (hierarchical clustering, UPGMA method) of *Amaranthus* taxa investigated. Main clades: (A) = *crassipes*-group (light green); (B) = *muricatus*/*viridis*-group (blue); (C) = residual-group (square with red sides). Secondary clades: *centralis*-group (orange), *graecizans*-group (violet), *induratus*-group (red), *retroflexus*-group (yellow), *vulgatissimus*-group (dark green).

The selection of *Amaranthus* taxa primarily depended on the availability of the Directors/Curators of the Herbaria to send material and on the occurrence of seeds in the exsiccata, which was made in a way to (1) cover all the continents (Figure 1) and (2) choice, for each taxon, specimens collected in distribution areas as wide as possible (Appendix A). The list of the *Amaranthus* taxa selected is shown in Table 1.

The identification of the species was made by using a stereomicroscope LEICA EZ4W and following literature [1,3,42]. The Nomenclature of the names follows [41], except for *Amaranthus emarginatus* Salzm. ex Uline & Bray (recognized as a subspecies of *A. blitum* L. by [41]) that is here accepted at species rank according to [43].

### 2.2. Scanning Electron Microscopic Analyses

Micro-morphological seed traits were examined by a scanning electron microscope SEM (JSM5910, 3 kv voltage, and secondary electron detector). The seeds for each *Amaranthus* species were mounted on metallic stubs using double adhesive tape and coated with gold for 6 min in a sputtering chamber followed by observation under SEM. The photographs were taken using different magnifications (from 50× to 10,000×) depending on the size of the seeds. For each seed, SEM micrographs were taken in lateral, frontal, and apical views, and on the hilum region.

### 2.3. Morphometric Analysis

In total, 25 taxa, up to 5 sites per taxon, and up to 5 seeds per sample (mature and not deformed/broken) were studied (Table 1). In total, 14 characters [7 qualitative, 7 quantitative (Table 2)] were measured on 111 samples (a total of 1554 measurements) using a scanning electron microscope SEM (JSM5910, 3 kv voltage, and secondary electron detector). Definitions of qualitative characters (excepting color) with associated types-images are reported in Table 3.

The data matrix (samples × variables) was processed using the software NCSS 2007. The variability of the characters has been examined by cluster analysis (UPGMA method), principal component analysis (PCA), discriminant analysis (DA), and box plots [in yellow boxes presented, illustrate interquartile ranges (=the range between the 25th and 75th percentile) and medians (horizontal line); vertical lines are the whiskers that represent the scores outside the middle 50% (i.e., the lower 25% of scores and the upper 25% of scores)]. PCA analysis was performed both by excluding the qualitative characters and by including them as binary variables according to [44]. DA was performed using the first six components derived from PCA, which explains about 69% of the total variability. The use of component scores (each other linearly independent by construction) allows obtaining an unbiased discriminant model both solving the indeterminacy due to the multicollinearity of the independent variables and receiving a more reliable prediction for the smaller number of involved variables [45,46,47]. We performed the DA on groups classified using both names of taxa (subgenera and species and below ranks) and major groups as a result of the molecular analyses by Waselkov et al. [15] (pag. 446, Figure 1A,B; hereafter reported as “molecular clades”). A k-means procedure (which is the most common unsupervised non-hierarchical clustering technique maximizing the between/within-cluster variance ratio (F-Ratio) for a given k number of clusters [48], was performed to identify the optimum number of groups without using “no prior knowledge”.

Concerning qualitative variables (nominal), we also prepared a matrix including the percentage of each variable for each taxon.

## 3. Results and Discussion

As a whole, the results of the morphometric analyses revealed that seeds micromorphology provides some new interesting taxonomic data concerning some taxa; on the other hand, others seed features are not useful for other species.

### 3.1. Analyses of the Whole Dataset

Hierarchical clustering (UPGMA method) shows three main groups. The first one is very small (A), including only samples of *Amaranthus crassipes*, a second small group (B) comprising samples of *A. viridis* and *A. muricatus*, and a third large group (C), with all the other samples/taxa (Figure 2). In group (C), several taxa (e.g., *A. albus*, *A. hybridus*, or *A. hypochondriacus*) are not grouped together, whereas some (*A. centralis*, *A. graecizans* subsp. *sylvestris*, *A. induratus*, *A. retroflexus*, and *A. vulgatissimus*) have a very low dissimilarity with each other and form separated subclades.

The three groups shown in Figure 2 do not correspond to the three subgenera of *Amaranthus* proposed by Mosyakin & Robertson [14] since clades (A) and (B) would be both parts of the subgen. Albersia, whereas in the large clade (C), all three subgenera (Acnida, Albersia, and Amaranthus) are represented and intermixed.

**Figure 3:**
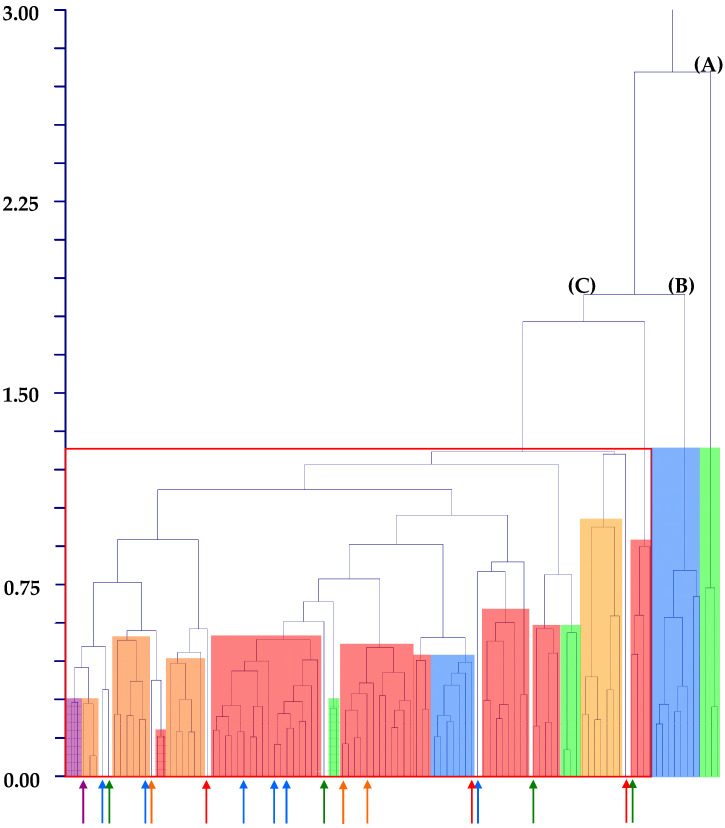
Dendrogram graph (hierarchical clustering, UPGMA method) of “molecular clades” *sensu* Waselkov et al. [15] (pag. 446, Figure 1A). Main clades: (A) = *Galápagos*-clade (light green); (B) = *South American*-clade (blue); (C) = residual-group (square with red sides); *Hybridus*-clade in red, *Eurasian/South African/Australian* (ESA)-clade in orange, *Dioecious/Pumilus*-clade in violet. Arrows indicate single samples of the various clades (colors are the same as those indicated in this caption), which are included in different clades or form isolated lineages.

**Figure 4:**
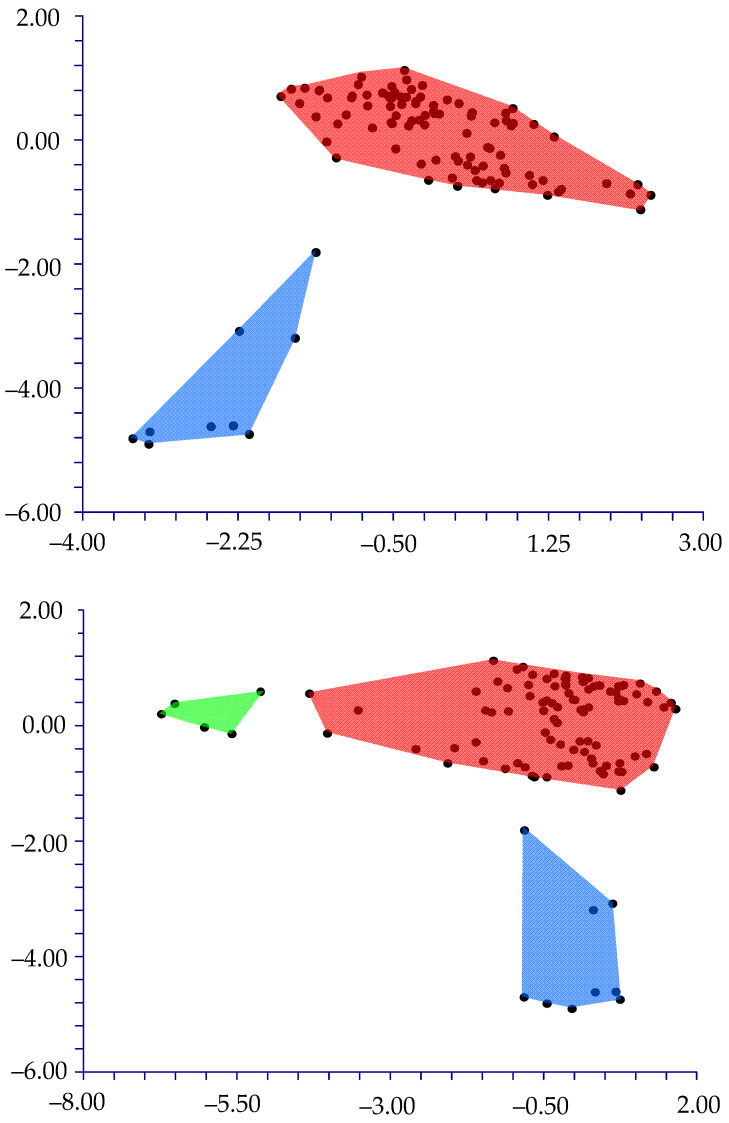
PCA graph: first (*x*-axis) vs. second (*y*-axis) components (graph on the top), and first (*x*-axis) vs. sixth (*y*-axis) components (graph on the bottom). Blue polygon: *muricatus*/*viridis*-group; green polygon: *crassipes*-group; red polygon: other samples/taxa.

The hierarchical clustering (UPGMA method) performed on Waselkov’s “molecular clades” [15] (note that all these Waselkov’s clades are represented in our samples) showed that no well-defined and separated group can be identified (Figure 3). Note that the dendrogram reported in Figure 4 refers to the molecular tree by Waselkov et al. [15] constructed based on nuclear genes. Hierarchical clustering performed on Waselkov’s “molecular clades” referred to chloroplast regions (not showed) reveals the same results, i.e., the absence of well-distinct groups.

The PCA shows that the cumulative percentage of eigenvalues for the first six axes is 68.86%, with a higher contribution (more than 10%) given by the first four components (17.96%, 15.51%, 11.28%, and 10.33%, respectively). Examining the combined graphs among pairs of these six components shows three well-separated groups (Figure 4) along the first and second components for the *muricatus*/*viridis*-group and the sixth component for the *crassipes*-group. The highest contributions to axes were given by the following characteristics: seed coat ornamentation, the central cell’s shape, and the flat border’s length.

**Figure 5:**
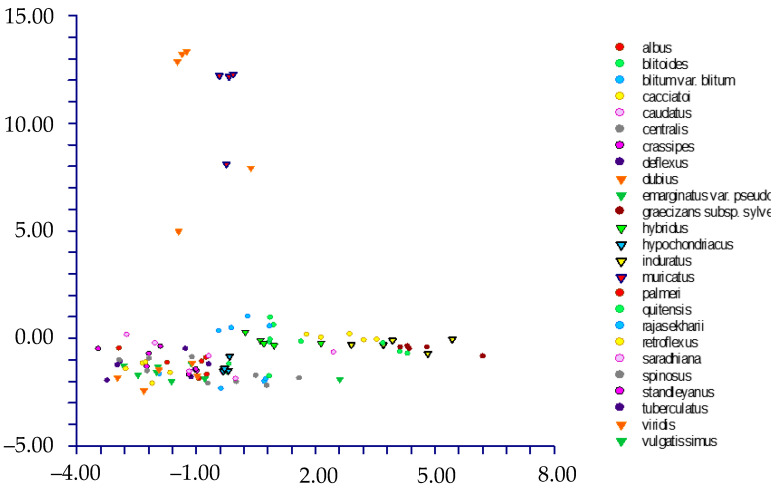
DA graph [first (axis-*x*) vs. second (axis-*y*) component] performed on samples classified using the taxa (species and below ranks) names.

**Figure 6:**
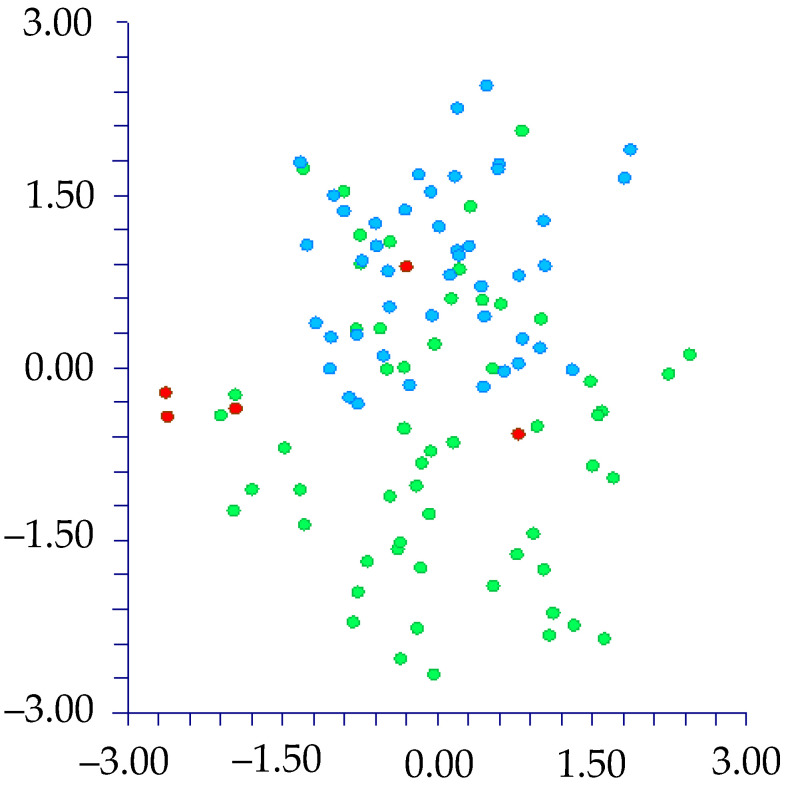
DA graph [first (axis-*x*) vs. second (axis-*y*) component] performed on samples classified using the subgenera names (blue dots: subgen. Amaranthus; red dots: subgen. Acnida; green dots: subgen. Albersia).

**Figure 7:**
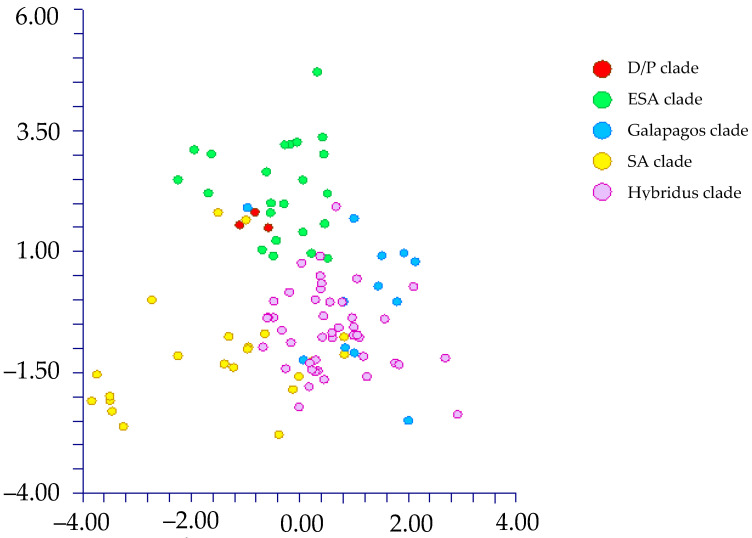
DA graph: [first (axis-*x*) vs. second (axis-*y*) component] performed on samples classified using Waselkov’s “molecular clades” [15] (nuclear genes).

The DA shows different results depending on the use of the names of the taxa (species and below ranks or subgenera) or Waselkov’s “molecular clades” [15]:

(1) By classifying the samples using the taxa names [species and below ranks (25 groups); see Table 1], DA predicted two main groups (Figure 5) based on the first two discriminant functions, which explain 71.3% of the total variation [eigenvalues: 52.2% (first function) and 19.1% (second function)]. These two groups (not overlapping each other) correspond to (a) the *muricatus*/*viridis*-group and (b) a large group including the remaining taxa (more or less overlapped each other). Concerning the *muricatus*/*viridis*-group, the matrix of actual/predicted groups displays high percentages along the diagonal (whose values reveal the matching actual/predicted observations for each group) for *Amaranthus viridis* (100%). In contrast, 50% of the actual observations for *A. muricatus* are predicted under *A. viridis*. Regarding the residual group, just a few taxa have actual/predicted observations matching each other (100%): *A. centralis*, *A. graecizans* subsp. *sylvestris*, *A. induratus*, *A. rajasekharii*, *A. spinosus*, and *A. tuberculatus*. On the contrary, low or very low percentages characterized the diagonal values of the other taxa, and, for *A. caudatus*, *A. dubius*, *A. hypochondriacus*, *A. palmeri*, and *A. standleyanus*, percentages are even zero. The value of correct classification is low (55.8%);
(2) When we classified the samples using the subgenera names (=three groups, i.e., *Acnida*, *Albersia*, and *Amaranthus*), DA do not predict any separate group, and the three groups completely overlapped each other (Figure 6). The first two discriminant functions explain 100% of the total variation [eigenvalues: 86.0% (first function) and 14.0% (second function)]. The matrix of actual/predicted groups reveals that (a) all the actual samples included in the Acnida-group are predicted as included in the other two groups (80% in the Albersia-group, 20% in the Amaranthus-group) and (b) about the 25% of the actual observations of the Albersia- and Amaranthus-groups is predicted under, respectively, the Amaranthus- and Albersia-groups.
  As a whole, the value of correct classification is low (57.5%);
(3) After running the DA procedure on samples classified using Waselkov’s “molecular clades” [15] (pag. 446, Figure 1A, based on nuclear genes), two groups were predicted (Figure 7) based on the first two discriminant functions, which explain 88.5% of the total variation [eigenvalues: 58.8% (first function) and 29.7% (second function)]. These two groups correspond to (A) the *Dioecious*/*Pumilus*-group+ESA-group (=Eurasian/S-African/Australian group [15]) and (B) a larger group including the remaining “molecular clades” *sensu* Waselkov et al. [15]. Note that the ESA-group partially overlaps the residual-group. The matrix of actual/predicted groups displays high percentages along the diagonal (whose values reveal the matching of actual and predicted observations for each group) for the ESA-group (96%) and *Hybridus*-group (95.65%), whereas low percentages characterized by the diagonal values of the *Galápagos*-clade [16.67%, whereas most of the percentage (66.67%) is predicted under the *Hybridus*-group], the *South American*-clade [45.45%, whereas the 45.45% is predicted under the *Hybridus*-group); finally, concerning the *Dioecious*/*Pumilus*-group, the 100% of the actual samples are predicted under the ESA-group. As a whole, the value of correct classification is low (64.6%).
  DA (not shown) performed on Waselkov’s “molecular clades” referred to chloroplast regions reveals the absence of separated groups. In fact, all the samples are intermixed among the various “molecular clades”.

**Figure 8:**
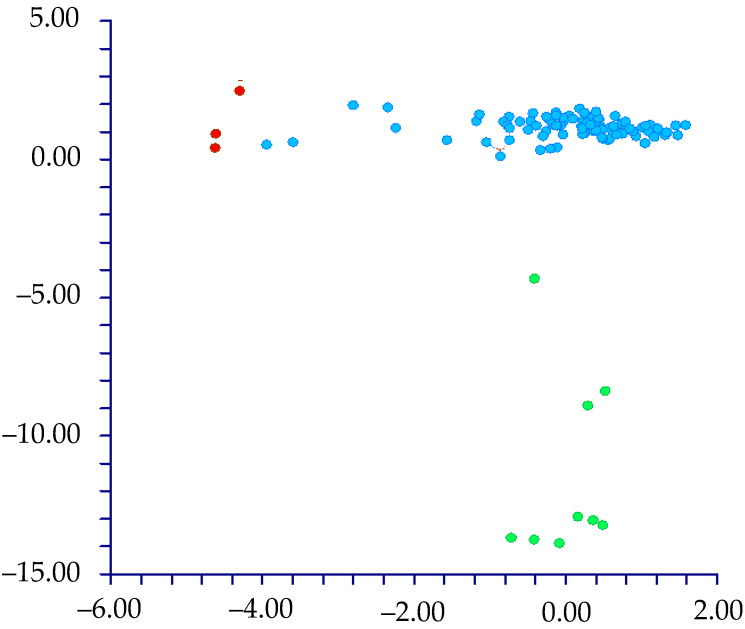
DA graph: [first (axis *x*) vs. second (axis *y*) component] performed on groups derived from PCA: red dots: *crassipes* group; green dots: *muricatus*/*viridis* group; blue dots: residual group.

Furthermore, we performed the DA using the three groups generated from the PCA (*muricatus*/*viridis*-group, *crassipes*-group, residual-group). The result is that these three groups are statistically well supported, based on the two discriminant functions, which explain 100% of the total variation [eigenvalues: 97.4% (first function), and 2.6% (second function)] (Figure 8). The value of correct classification is high (94.3%).

K-means confirm the three clusters solution for the samples considered showing a high F-Ratio (197.13) of the first PCA component (which gives the higher contribution in PCA analysis, i.e., 17.96%) in the 3-clustered running procedure; in contrast, F-Ratios are 185.30, 132.16, and 99.9 in, respectively, 2-, 4-, and 5-clustered procedures. (Table 4).

**Figure 9:**
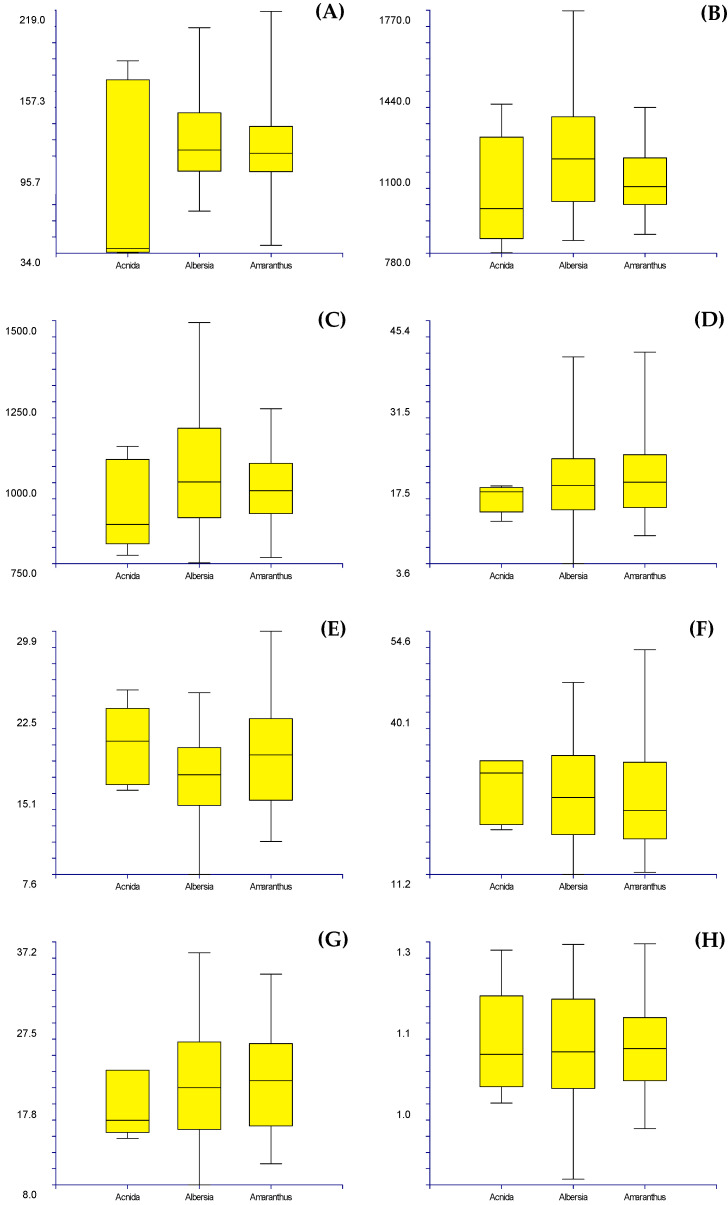
Box plots illustrating the variability of the quantitative micromorphological seeds characteristics (*y*-axes) per subgenera (*x*-axes): length of the flat border (**A**), length of the seed (**B**), width of the seed (**C**), the average length of the peripheral cells (**D**), the average width of the peripheral cells (**E**), the average length of the central cells (**F**), the average width of the central cells (**G**), ratio length/width of the seeds (**H**). Measurements are in µm.

**Figure 10:**
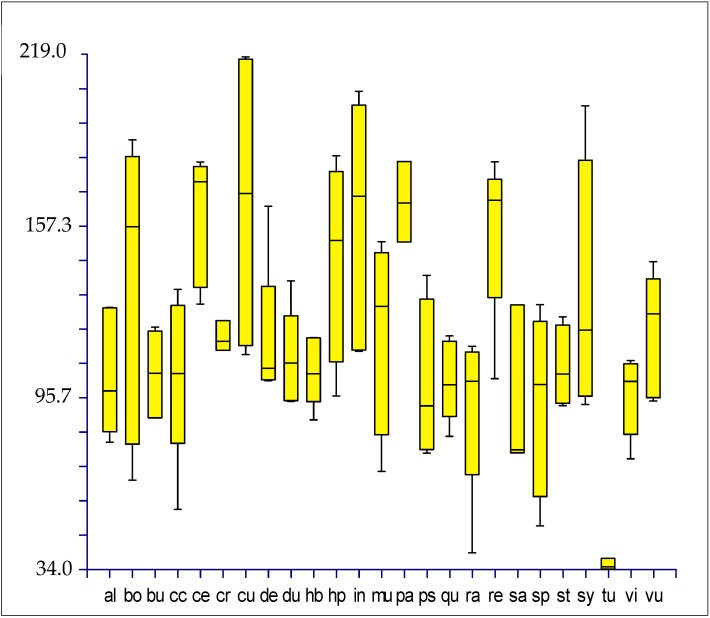
Box plots illustrating the variability of the length of the flat border of the seed (*y*-axis; measurements are in µm) per taxon (*x*-axis; abbreviation follow Table 1).

**Figure 11:**
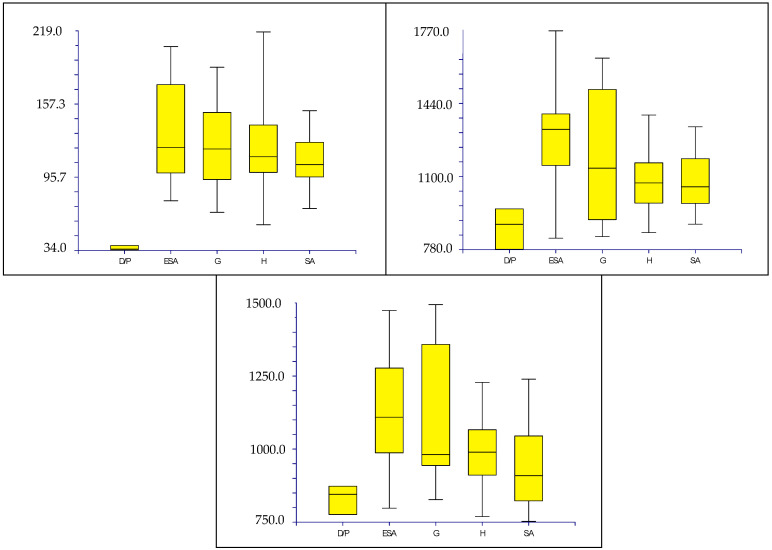
Box plots [characters (*y*-axes) per “molecular clades” (*x*-axes)] illustrating the variability of the length of flat border opposite to hilum (left graph), length of seed (central graph), and width of seed (right graph) (measurements are in µm). Abbreviation: D/P = *Dioecius*/*Pumilus*-clade; ESA = *Eurasian/South African/Australian*-clade; G = *Galápagos*-clade; SA = *South American*-clade; H = *Hybridus*-clade [clades according to Waselkov et al. [15] (pag. 446, Figure 1A].

Box plots, made on quantitative characters (see Table 2), show the following results:

(1) Subgenus rank (*sensu* Mosyakin & Robertson [14]): no group can be distinguished using seed micromorphology (Figure 9).
(2) Species and below ranks: only one species (*Amaranthus tuberculatus*) can be clearly distinguished from all the other ones by using the length of the flat border of the seed, which is very small [34–38 µm vs. (40.01–)66.04–214.67(–218.07) µm] (Figure 10);
(3) “Molecular clades” [*sensu* Waselkov et al. [15] (pag. 446, Figure 1A, based on nuclear genes)]: only the *Dioecious*/*Pumilus*-group can be distinguished based on micromorphology of seeds, i.e., by the length of the flat border of the seed and, partially, the length and width of the whole seed (Figure 11).
(4) Box plots (not shown) originated based on Waselkov’s “molecular clades,” which refer to chloroplast regions that do not reveal any separate group.

Concerning the qualitative characters (nominal variables; see Table 2), the synoptical matrix of taxa confirms the three main groups resulting from hierarchical clustering, PCA, and DA analyses. These groups can be distinguished based on seed coat ornamentation. The *muricatus*/*viridis*-group has a wrinkled coat, the *crassipes*-group shows a pebble-stoned coat, and the residual-group displays a puncticulate or colliculate coat (never wrinkled or pebble stoned). The shape of the central cells also allows for distinguishing the *muricatus*/*viridis*-group, which included taxa showing about 80% of seeds with an irregular shape. In contrast, the other two groups (*crassipes* and the residual group) have central cells with a regular shape (about 100%). Finally, no character is useful to distinguish groups using both subgenera and “molecular clades” classifications.

### 3.2. Analyses on Subdatasets

As stated in the previous paragraph, samples referred to some taxa, despite being included in the large so-called “residual-group” (see Figure 4 and Figure 5), have a very low dissimilarity with each other in the hierarchical clustering procedure and form separated subclades. These taxa are *A. centralis*, *A. graecizans* subsp. *sylvestris*, *A. induratus*, *A. retroflexus*, and *A. vulgatissimus*. So, we try to understand if differential combinations of seed characters in comparison with the related taxa characterized them as follows:

➢ *Amaranthus centralis*: it is an endemic Australian species (Northern Territory, Queensland, South Australia, and Western Australia) macro-morphologically similar to *A. induratus*. According to Palmer [42], these two species differ from each other based on the shape of the leaves (linear to narrowly oblong or narrowly ovate in *A. induratus* vs. ovate or elliptic in *A. centralis*) and tepals (margins with a single or serrated tooth-like projection on each side vs. margins without tooth-like projections). Note that *A. centralis* and *A. induratus* are included in the same Waselkov’s “molecular clade,” i.e., the ESA-clade.” (ESA = Eurasian/S-African/Australian group [15]). Moreover, our analyses reveal a micro-morphological similarity between these two taxa as follows:
  > In the hierarchical clustering procedure (UPGMA method), the *centralis*-group and *induratus*-group are part of the same subgroup of the large “residual-group” (see Figure 3);
  > In K-means 10-clustered procedure (not shown), one of the clusters is composed of the samples of *A. centralis* and *A. induratus*;
  > In DA analysis performed on samples classified using taxa, actual/predicted observations match each other for these two species (100% percentages along the diagonal).

Box plots show that *Amaranthus centralis* and *A. induratus* differ from each other by the seed length [(1.18–)1.21–1.34(–1.38) mm vs. (1.36–)1.39–1.51(–1.77) mm] and width [(0.91–)1.01–1.02(–1.04) mm vs. (1.11–)1.17–1.18(–1.47) mm] and their ratio (1.31–1.33 mm vs. 1.19–1.28 mm). In contrast, qualitative differential features are the seed shape (usually oval in *Amaranthus centralis* vs. usually circular in *A. induratus*) and coat ornamentation (puncticulate vs. colliculate).

➢ *Amaranthus graecizans* subsp. *sylvestris*: it is a taxon native to central and southern Europe and north Africa, and it is (macro-) morphologically characterized by having leaves usually acute, flowers arranged in axillary glomerules, three tepals in pistillate flowers, and fruit as long as or longer than the perianth [3]. This morphological configuration is similar to the forms of the Asian *A. tricolor* L. without terminal synflorescences [3,7,49], which were originally published by Linnaeus as *A. tristis* L. [50], *A. tricolor* [50], and *A. polygamus* L. [51], but later synonymized with *A. tricolor* [52]. *A. graecizans* subsp. *sylvestris* is included in the ESA-clade by Waselkov et al. [15], where also *A. tricolor* occurs. *A. graecizans* subsp. *sylvestris* and *A. tricolor* can be distinguished using seed micromorphology by the seed length [(1.09–)1.28–1.34(–1.47) vs. (0.20–)0.90–1.26], the seed width [(1.05–)1.20–1.36] vs. 0.97–1.09(–1.11)], seed shape (circular vs. oval), and seed type (not reticulate vs. reticulate);
➢ *Amaranthus retroflexus*: it is native to Mexico and alien in all other continents where it is often widely spread [41]. This species can be distinguished by its stem, usually tomentose-pubescent and erect, green synflorescence, and five tepals spathulate with obtuse to emarginate apex [1,3,7,53]. *A. retroflexus* is macromorphologically similar to *A. wrightii* S.Watson, which differs mainly by being glabrous or nearly so [1,7]. Other authors e.g., [53] highlighted the similarity between *A. retroflexus* and *A. quitensis* (Bolòs & Vigo [54] even proposed to treat *A. quitensis* as subspecies of *A. retroflexus*). All these three species (*A. quitensis*, *A. retroflexus*, and *A. wrightii*) belong to the *Hybridus*-clade *sensu* Waselkov et al. [15]. Our analyses show that *A. retroflexus* differs from *A. quitensis* by the length of seed flat border [(102.53–)160.70–167.86(–180.35) µm vs. (81.8–)96.08–114.05(117–87) µm], the seed length [(1.04–)1.17–1.21 mm vs. (1.04–)1.06–1.11(–1.12) mm], the ratio length/width [(1.09–)1.10–1.14(–1.16) vs. (0.97–)0.99–1.06(–1.09)], and the seed type (not reticulate vs. reticulate);
➢ *Amaranthus vulgatissimus*: it is a species native to South America (Argentina and Uruguay) that appears well morphologically distinct among the taxa with five tepals and indehiscent fruits [*A. cochleitepalus* Domin, *A. crispus* (Lesp. & Thévenau) A.Braun ex J.M.Coult. & S.Watson, *A. cuspidifolius* Domin, and *A. persimilis* Hunz.) having tepals oblong with a base 0.3–0.5 mm wide (vs. spathulate with a base up to 0.3 mm wide in the other four mentioned species) [7]. In Waselkov et al. [15], *A. vulgatissimus* belongs to the SA-clade, sister to *A. muricatus*. The latter species was part of the *muricatus*/*viridis*-group according to our analyses (see Figure 2, Figure 4, Figure 5 and Figure 8), and it differs primarily by the seed coat ornamentation (wrinkled) and shape of the central cell (irregular), whereas *A. vulgatissimus* has colliculate seed coat and regular central cells. A further difference between these two species refers to the width of the seed [(0.84–)0.85–0.99(–1.07) vs. (1.11–)1.14–1.16(–1.24)].

In addition to the above-discussed species, *Amaranthus rajasekharii*, *A. spinosus*, and *A. tuberculatus* can also be analyzed in detail by considering the results of DA using the names of the taxa. As highlighted in the previous paragraph, the actual/predicted observations for these three species match each other (100%). *A. tuberculatus* is discussed above (see paragraph “3.1. Analyses on the whole dataset”); some comments about the other two species and the related ones as follows:

➢ *Amaranthus rajasekharii*: a species recently described from India [55] morphologically related to *A. dibius* Mart. from which differ by the stem (reddish to purple vs. green in *A. dubius*), bracts (linear and up to 0.1 mm long vs. ovato-deltoid, 1.3–1.7 mm long), tepals shape (ovate to lanceolate vs. oblong-spatulate), gynoecium (whitish vs. green), and pollen grain [with 21–23 pores (vs. 27–30), 3–5 ektexinous bodies (vs. mostly 3), and margin of pores not depressed and without conspicuous ornamentation (vs. clearly depressed and with conspicuous ornamentation)]. *A. dubius* is included in the *Hybridus*-clade by Waselkov et al. [15], whereas *A. rajasekharii* did not appear in their work being published later. Our results reveal that these two species differ from each other by the ratio length/width of seeds [(0.98–)1.03–1.05(–1.11) in *A. rajasekharii vs*. (1.09–)1.11–1.14(–1.26) in *A. dubius*];
➢ *Amaranthus spinosus*: a species native to tropical America (from Mexico to Argentina), which is easy to distinguish from the other ones by its spine-like structure (metamorphosed bracts of the first flower in the first cyme) [1,3,7,53]. Recently, a similar species (*A. saradhiana* Sindhu Arya, V.S.A.Kumar, W.K.Vishnu & Rajesh Kumar) was described from India. It has, along the stem, only two spines per node, whereas no spines occur in the synflorescence part (4 spines per node along the stem and spines in the synflorescence part in *A. spinosus*); further differences regard the stem and petiole color (purple vs. green in *A. spinosus*), the apex of tepals (acute vs. often spathulate), gynoecium (whitish to light green vs. dark green), and the number of pores in pollen grains (26–30 vs. 37–40). *A. spinosus* is included in the *Hybridus*-clade by Waselkov et al. [15], whereas *A. saradhiana* did not appear in their work, being published later. Our results show that these two species differ from each other by the seed length [0.80–0.89(–0.94) vs. 0.98–1.02 in *A. spinosus*], seed width [(0.80–)0.98–1.04(–1.10) vs. 0.99–1.02], and peripheral cells length [20.00–28.05 vs. (8.39–)11.48–16.31(–17.88)], whereas, concerning the qualitative seed characters, some are partially overlapped [color (mostly reddish brown vs. dark brown), coat ornamentation (mostly punticulate vs. colliculate), type (mostly not reticulate vs. reticulate), shape of peripheral cells (polygonal vs. mostly tetragonal), pleurogram (present vs. sometimes absent)].

### 3.3. Seed Types and Taxonomic Key

The deep morphometric analyses on seeds micromorphology show that several seed characters are taxonomically useful to distinguish some taxa both by considering the whole dataset (25 taxa) and by comparing taxa related from morphological and/or molecular points of view. On the contrary, seed characters are not able to separate groups at a rank higher than species (subgenus), so confirming the molecular results by Waselkov et al. [15] (pag. 446, Figure 1A,B). Moreover, seed characters analysis does not allow to distinguish Waselkov’s “molecular clades,” except for the *Dioecious*/*Pumilus*-group (nuclear genes), which is distinguished by the length of the flat border of the seed and, partially, the length and width of the whole seed (see Figure 11).

The morphological differences found (especially those statistically well supported) allow us to define some seed types. On the contrary, in other cases, we prefer to refrain from proposing defined types for the moment. A tentative taxonomic key of the studied taxa/seed types, based on micromorphological characters, is below presented. Concerning the seeds coat ornamentation dichotomous alternatives at the step no. 4 of the key (punticulate vs. colliculate), we introduced the term “mostly” since (1) type punticulate always occurs in three taxa (*A. centralis*, *A. emarginatus* subsp. *pseudogracilis*, and *A. graecizans* subsp. *sylvestris*), whereas *A. albus*, *A. blitum*, and *A. sarahdiana* have seeds mostly punticulate (up to 80%); (2) type colliculate is typical (100% of the studied samples) of eight taxa (*A. deflexus*, *A. palmeri*, *A. hybridus*, *A. hypochondriacus*, *A. induratus*, *A. quitensis*, *A. retroflexus*, *A. spinosus*, and *A. vulgatissimus*), whereas *A. blitoides*, *A. cacciatoi*, *A. caudatus*, *A. dubius*, and *A. rajasekarii* have seed mostly punticulate (up to 80%); *A. standleaynus*, which shows 50% of seeds punticulate and the rest colliculate, was included under two parts of the key (steps nos. 5–9 and 10–19). Finally, note that *A. deflexus*, *A. vulgatissimus*, *A. cacciatoi*, *A. spinosus*, *A. dubius*, and *A. stadleyanus* (which we classified in the same seed *deflexus*-type) cannot be distinguished using seeds characters.

1. Seed coat ornamentation pebble stoned ................................. ***crassipes*-type** (*A. crassipes*)
1. Seed coat ornamentation never pebble stoned .................................................................... 2
2. Seed coat ornamentation wrinkled ..................................................................... ***viridis*-type**
  2a. Seed black, (0.89–)0.95–1.04(–1.06) mm long, (0.82–)0.87–0.95(–1.04) mm wide, pleurogram often absent .................................................................................... *A. viridis*
  2b. Seed dark-brown, (1.24–)1.26–1.33 mm long, (1.11–)1.14-1.16(–1.24) mm wide, pleurogram present ...................................................................................... *A. muricatus*
2. Seed coat ornamentation never wrinkled ............................................................................. 3
3. Flat border of the seed 34.14–38.04 µm long ............... ***tuberculatus*-type** (*A. tuberculatus*)
3. Flat border of the seed longer ................................................................................................. 4
4. Seed coat ornamentation mostly puncticulate ..................................................................... 5
4. Seed coat ornamentation mostly colliculate ....................................................................... 10
5. Peripheral cells polygonal, seed mostly reddish-brown ............................... *A. sarahdiana*
5. Peripheral cells tetragonal, seed dark-brown ...................................................................... 6
6. Seed ratio length/width 1.17–1.33 ........................................................................................... 7
6. Seed ratio length/width smaller ............................................................................................. 8
7. Seed circular, 0.99–1.12(–1.18) mm long, ratio length/width (1.17–)1.20–1.22; flat border of the seed (92.82–)96.36–112.90(–124.65) µm long ....................................... *A. stadleyanus*
7. Seed mostly oval, (1.18–)1.21–1.32(–1.39) mm long, ratio length/width 1.31–1.33; flat border of the seed (129.28–)141.30–176.98(–180.30) µm long ........................... *A. centralis*
8. Seed mostly not reticulate ......................................................................................................... 9
9. Seed (1.09–)1.28–1.34(–1.47) mm long, (1.05–)1.20–1.24(–1.36) mm wide........................................................................................... *A. graecizans* subsp. *sylvestris*
9. Seed (0.84–)0.86–0.89(–0.98) mm long, (0.83–)0.94–0.95(–0.98) mm wide................................................................................................................................ *A. albus*
8. Seed reticulate ......................................................................................................... ***blitum*-type**
  8a. Seed (0.95–)1.02–1.16(–1.21) mm long .......................................... *A. blitum* var. *blitum*
  8b. Seed 0.84–1.02 mm long ...................................... *A. emarginatus* subsp. *pseudogracilis*
10. Peripheral cells polygonal ................................................................................... *A. rajasekarii*
10. Peripheral cells tetragonal .................................................................................................... 11
11. Seed ratio length/width 1.17–1.33 ........................................................................................ 12
11. Seed ratio length/width smaller ........................................................................................... 17
12. Seed (1.36–)1.39–1.48(–1.77) mm long ................................................................ *A. induratus*
12. Seed shorter ............................................................................................................................ 13
13. Seed 1.25–1.39 mm long ......................................................................................................... 14
13. Seed shorter ............................................................................................................................ 15
14. Peripheral cells 16.38–16.94 mm long, central cells 13.61–15.86 mm wide............................................................................................................................. *A. palmeri*
14. Peripheral cells (15.44–)19.04–25.71(–33.15) mm long, central cells (15.25–)16.88–20.83(–22.71) mm wide ..................................................... *A. hypochondriacus*
15. Seed not reticulate ................................................................................................ *A. retroflexus*
15. Seed reticulate ........................................................................................................................ 16
16. Seed black ................................................................................................................. *A. hybridus*
16. Seed mostly dark-brown ................................................................................... ***deflexus*-type**

(*A. deflexus*, *A. vulgatissimus*, *A. cacciatoi*, *A. spinosus*, *A. dubius*, *A. stadleyanus*)

17. Seed 0.87–1.12 mm long ........................................................................................................ 18
17. Seed longer .............................................................................................................................. 19
18. Seed 0.87–0.91(–1.04) mm long, (0.77–)0.79–0.87(–0.94) mm wide ................... *A. spinosus*
18. Seed (1.02–)1.04–1.11(–1.12) mm long, 0.99–1.07 mm wide .............................. *A. quitensis*
19. Seed (1.13–)1.41-1.58(–1.64) mm long ................................................................... *A. blitoides*
19. Seed (0.99–)1.16-1.30 mm long .............................................................................. *A. caudatus*

**Figure 12:**
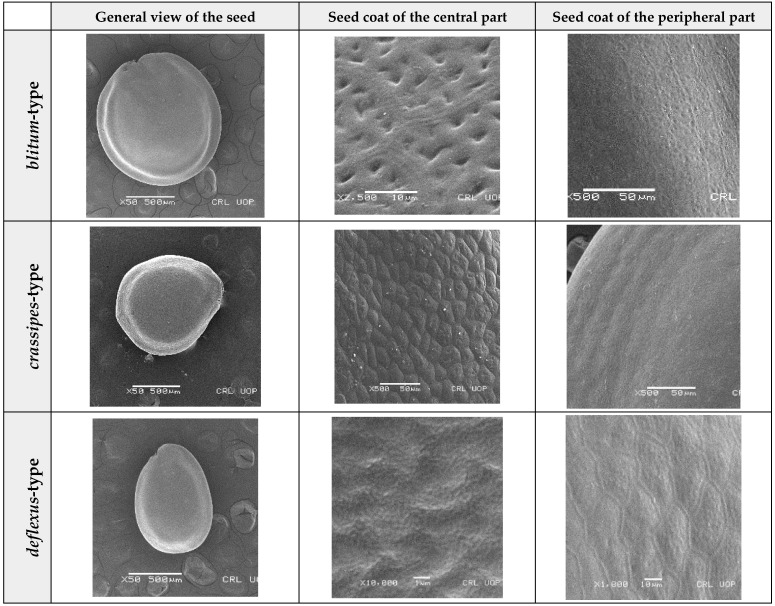

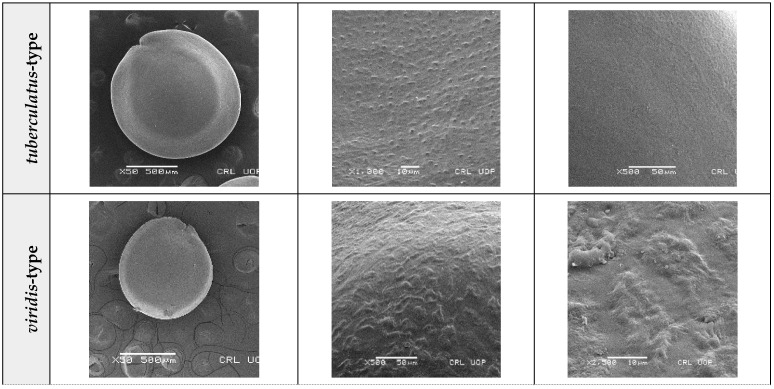
Types-pictures of the seed types defined.

Concerning the seed types defined, detailed descriptions follow (and see Figure 12):

(1) ***Blitum*-type**: seed (0.83)0.94–1.37(–1.42) × (0.80–)0.83–0.99(1.35) mm (length × width), circular, dark-brown, puncticulate, reticulate; ratio length/width (0.95–)1.00–1.04(–1.17); flat border (75.76–)80.93–120.97(–139.58) µm long; peripheral cells (3.58–)12.48–26.55(–29.11) × (7.61–)13.20–19.99(–20.95) µm on average, tetragonal; central cells (27.58–)27.99–38.59(–54.77) × (12.33–)13.36–25.97(–35.88) µm on average, mostly regular; pleurogram mostly present.
  **Species included:** *Amaranthus blitum*, *A. emarginatus* subsp. *pseudogracilis*.
(2) ***Crassipes*-type**: seed 1.04–12.0 × 0.92–1.04 mm (length × width), circular, dark-brown, pebble stone-like, reticulate; ratio length/width 1.13–1.23; flat border 112.71–123.36 µm long; peripheral cells 8.84–20.31 × 12.70–23.71 µm on average, tetragonal; central cells 25.40–28.18 × 16.23–24.19 µm on average, regular; pleurogram not present.
  **Species included:** *Amaranthus crassipes*.
(3) ***Deflexus*-type**: seed (0.87–)0.92–1.18(–1.24) × (0.75–)0.91–0.97(1.10) mm (length × width), oval or circular, mostly dark-brown, puncticulate or colliculate, reticulate; ratio length/width (0.98–)1.09–1.34(–1.41); flat border (49.66–)55.66–144.49(–164.43) µm long; peripheral cells (8.39–)10.51–39.16(–45.41) × (12.02–)12.90–24.10(–25.85) µm on average, tetragonal; central cells (13.85–)14.41–45.39(–51.24) × (10.58–)11.01–27.89(–28.43) µm, regular; pleurogram mostly present.
  **Species included:** *Amaranthus deflexus*, *A. vulgatissimus*, *A. cacciatoi*, *A. spinosus*, *A. dubius*, and *A. stadleyanus*.
(4) ***Tuberculatus*-type**: seed 0.78–0.86 × 0.78–0.87 mm (length × width), circular, dark-brown, puncticulate, mostly not reticulate; ratio length/width 1.01–1.10; flat border 34.14–38.04 µm long; peripheral cells 10.86–16.05 × 15.35–24.53 µm on average, tetragonal; central cells 19.14–31.47 × 15.04–21.79 µm on average, regular; pleurogram present.
  **Species included:** *Amaranthus tuberculatus*.
(5) ***Viridis*-type**: seed (0.89–)0.95–1.31(–1.33) × (0.82–)0.87–1.16(–1.24) mm (length × width), circular, dark-brown or black, wrinkled, reticulate; flat border (69.20)73.76–135.88(–151.69) µm long; peripheral cells (8.56–)8.84–19.87(–23.16) × (9.63–)11.00–20.75(–20.85) µm on average, tetragonal; central cells (11.16–)14.38–22.01(–38.95) × (8.04–)12.33–25.78(–30.49) µm on average, usually irregular; pleurogram present or not.
  **Species included:** *Amaranthus muricatus*, *A. viridis*.

## 4. Conclusions

Seed micromorphology was shown to be a highly informative taxonomic criterion that helps to solve ambiguities in plant taxonomy. Concerning the genus *Amaranthus*, few articles have been published until now, and they refer to one to a few species. Our paper is the first one, including a good number of species (25).

Both the results obtained and the proposed diagnostic key highlight, on the one hand, the seed micromorphology can be considered a useful tool in the taxonomy of *Amaranthus*, but, on the other hand, various species cannot be distinguished using these types of features (e.g., the so-called “*deflexus*-type”, comprising *A. deflexus*, *A. vulgatissimus*, *A. cacciatoi*, *A. spinosus*, *A. dubius*, and *A. stadleyanus*). These facts reveal, once again, the taxonomic complexity of the genus *Amaranthus* as already highlighted by many authors [1,2,3,5,6,7,8,9,10,11,12,13,14,15,16,32,33,41,43,49,52,53]. Moreover, based on the studies, taxa, just some seed types, can be defined. Therefore, we argue for future investigations on seed micromorphology by adding new species. A suggestion could be to study all the taxa belonging to the most critical groups, e.g., the *hybridus* group (*sensu* Waselkov et al. [15]), the *blitum* group (*sensu* Iamonico & Das [43], or the *graecizans* group [3,56].

## Figures and Tables

**Table 1:** List of the *Amaranthus* taxa studied (alphabetical order). Native distribution areas from [41] and, concerning *A. blitum*, from [3]. S: South; W: West; N: North: E: East; C: Central.

Code	Taxon	Native Distribution Area
al	*Amaranthus albus* L.	S-U.S.A. to NE-Mexico
bo	*Amaranthus blitoides* S.Watson	CE-U.S.A.
bu	*Amaranthus blitum* L.	Mediterranean area and C-Europe
cc	*Amaranthus cacciotoi* (Aellen ex Cacciato) Iamonico	C-Italy
ce	*Amaranthus caudatus* L.	Ecuador to NW-Argentina
cr	*Amaranthus centralis* J.Palmer & Mowatt.	Australia
cu	*Amaranthus crassipes* Schltdl.	U.S.A. to N-Mexico, Caribbean, S-America
de	*Amaranthus deflexus* L.	S-America
du	*Amaranthus dubius* Mart. ex Thell.	S-America
hb	*Amaranthus emarginatus* Salzm. ex Uline & Bray	Brazil to N-Argentina
hp	*Amaranthus graecizans* subsp. *sylvestris* L.	CS-Europe and N-Africa
in	*Amaranthus hybridus* L.	NC-America
mu	*Amaranthus hypochondriacus* L.	C-U.S.A. to Mexico
pa	*Amaranthus induratus* J.Palmer & Mowatt.	N-Australia
ps	*Amaranthus muricatus* (Gillies ex Moq.) Hieron.	S-America
qu	*Amaranthus palmeri* S.Watson.	S-California to Texas and Mexico
ra	*Amaranthus quitensis* Kunth.	S-America
re	*Amaranthus rajasekharii* Sindu Arya et al.	India
sa	*Amaranthus retroflexus* L.	N-America
sp	*Amaranthus saradhiana* Sindu Arya et al.	India
st	*Amaranthus spinosus* L.	Mexico to Tropical America
sy	*Amaranthus standleyanus* Parodi ex Covas.	S-America
tu	*Amaranthus tuberculatus* (Moq) J.D.Sauer	EC-U.S.A.
vi	*Amaranthus viridis* L.	Mexico to Tropical America
vu	*Amaranthus vulgatissimus* Speg.	S-America

**Table 2:** Characters measured for the morphometric analysis. The characters labelled with an asterisk (*) are qualitative, and the others are quantitative (see Table 3).

1. Seed length (mm)
2. Seed width (mm)
3. Length of flat border opposite to hilum (µm)
4. Average peripheral cells length (µm)
5. Average peripheral cells width (µm)
6. Average central cells length (µm)
7. Average central cells width (µm)
8. Seed color (reddish-brown, dark-brown, black) *
9. Seed shape (circular, not circular) *
10. Seed coat ornamentation (puncticulate, colliculate, pebble stone-like, wrinkled) *
11. Seed reticulation (reticulate, not reticulate) *
12. Peripheral cells shape (tetragonal, polygonal) *
13. Central cells shape (regular, irregular) *
14. Pleurogram (present, not present) *

**Table 3:** Qualitative characters, their definition, and associated types-pictures.

Seed Coat Ornamentation			Seed Shape		Seed Reticulation	
*Puncticulate*: a surface that has fine and widely spaced punctures 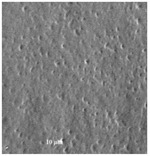			*Circular*: ratio length/width ≈ 1 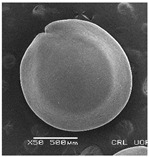		*Reticulate*: a surface that have a network of lines 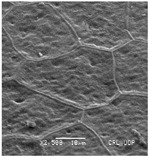	
*Colliculate*: a surface that has low, rounded elevations 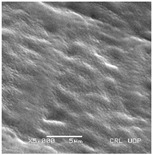			*Not circular*: ratio length/width > 1 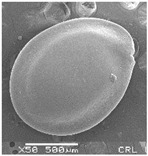		*Not reticulate*: a surface without a network or network unclear 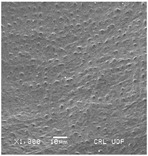	
*Pebble stone-like*: a surface that has ± rounded like-stones structures 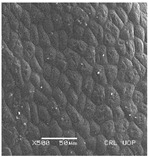			**Peripheral cells shape**		**Central cells shape**	
			*Tetragonal*: cells with four sides 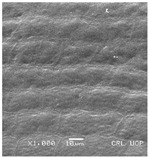		*Regular*: cells with distinct boundaries 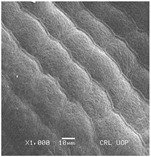	
*Wrinkled*: a surface that has slight folds or creases and crumples 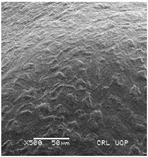			*Polygonal*: cells with > 5 sides 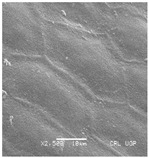		*Irregular*: cells without clear boundaries 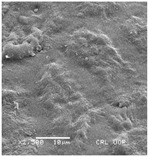	
	**Pleurogram**	*Present* 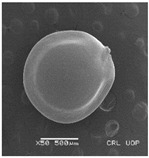		*Not present* 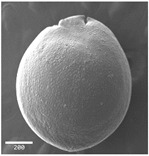		

**Table 4:** K-means procedure performed on 2, 3, 4, and 5 clusters.

No. of Clusters	Component	Between Mean Square	Within Mean Square	F-Ratio
2	1	73.46075	0.396	185.31
	2	25.385695	0.816	31.1
	3	0.1758203	1.008	0.17
	4	11.79855	0.988	11.94
	5	0.8752245	1.069	0.82
	6	1.435734	2.422	0.59
3	1	74.58856	0.3783803	197.13
	2	31.96614	0.6963916	45.9
	3	2.013769	0.9821099	2.05
	4	16.71954	0.9021953	18.53
	5	26.66274	0.5785692	46.08
	6	4.862731	2.378553	2.04
4	1	49.88319	0.377458	132.16
	2	21.86316	0.6869719	31.83
	3	18.39766	0.4901165	37.54
	4	20.30244	0.6417438	31.64
	5	16.79983	0.6129277	27.41
	6	1.237907	2.460811	0.5
5	1	37.54745	0.3758467	99.9
	2	17.43485	0.6526853	26.71
	3	14.16077	0.4806115	29.46
	4	15.74832	0.6274446	25.1
	5	12.90233	0.6070178	21.26
	6	47.03387	0.6592174	71.35

## Data Availability

Not applicable.

## Appendix A. List of Specimens Considered in the Study

Selected specimens collected from different herbaria’s or during the field surveys. The names of the taxa are reported in alphabetical order.

***Amaranthus albus* L.**

ROMANIA: Basarabia District Lapusna, cultivated and uncultivated land, 106 m a.s.l., 1930, *sine coll. s.n.* (RO No. 5928). ITALY: Latium, Ciampino, 126 m a.s.l., 22 June 2008, *sine coll. s.n.* (RO No. 4876). ISRAEL: Bet Shemesh, 264 m a.s.l., *s.d., D. Zohary & I. Amdusky 423,* (RO No. 5928). INDIA: Coimbatore Tamil Nadu, 430 m a.s.l., 27 October 2021, *A. Sindhu*, 254 (UCBD16). TUNISIA: Bizerte, 26 m a.s.l., 23 November 2018, *sine coll. s.n.* (RO).

***Amaranthus blitoides* S.Watson**

SPAIN: Barcelona, Manlleu, cultivated and uncultivated land, 466 m a.s.l., *s.d., sine coll. s.n.* (RO No. B972946). SAUDI ARABIA: Jizan, 5 m a.s.l., 17 February 2021, *sine coll. s.n.* (RO No. 2). TUNISIA: Bizerte, 18 m a.s.l., 23 November 2015, *sine coll. s.n.* (RO). BOSNIA-HERZEGOVINA: Zivinice, 208 m a.s.l., 30 September 2020, *S. Sarie, s.n.* (RO). ITALY: Calabria, Tropea, train station, 94 m a.s.l., 2007, *sine coll. s.n.* (HFLA).

***Amaranthus blitum* L. var. *blitum***

BOSNIA-HERZEGOVINA: Ribnica, Zavidovići, roadsides, shorelines, farmed lands, 306 m a.s.l., 30 September 2020, *S. Sarie, s.n.* (RO). ITALY: Latium, Rome, Arco di travertino, 41 m a.s.l., 15 June 2008, *D. Iamonico, s.n.* (RO No. 4881); Campania, Naples, Pepperoncino birichino, 4 m a.s.l., 21 July 2021, *A.N. Hussain 1*, (IS No. BL1); Molise, Isernia, Piazza San Pietro, 441 m a.s.l., 16 July 2021, *A.N. Hussain 2,* (IS No. BL2); INDIA: Kanchipuram Tamil Nadu, 300 m a.s.l., 27 October 2021, *A. Sindhu*, 260 (UCBD17).

***Amaranthus cacciotoi* (Aellen ex Cacciato) Iamonico**

ITALY: Latium, Rome, along the roads, vacant places, 16 August 1957, *A. Cacciato, s.n.* (RO); Rome, 20 September 1968, *A. Cacciato, s.n.* (RO); Rome, *s.d., A. Cacciato., s.n.* (RO No. 39); Rome, *s.d., sin coll., s.n.* (RO No. 58 Allen’s Number); Rome, 9 March 1960, *sin coll., s.n.* (RO).

***Amaranthus caudatus* L.**

SERBIA: Kragujevac, artificial habitat, 356 m a.s.l., 9 February 2019, *sin coll., s.n.* (RO). BOSNIA-HERZEGOVINA: Zivinice, 215 m a.s.l., 30 September 2020, *S. Sarie, s.n.* (RO). ROMANIA: Oravita, 215 m a.s.l., 9 January 2019, *sin coll., s.n.* (RO). INDIA: Chickpet, Karnataka, 320 m a.s.l., 21 December 2021, *Arya Sindhu,* 675 (UCBD25).

***Amaranthus centralis* J.Palmer & Mowatt**

AUSTRALIA: Tod River, Alice Spring, places with constant irrigation, bore-holes, parks, and agricultural lands, 586 m a.s.l., *s.d., sin coll., s.n.* (CANB33750); Nilpinna near Lake Eyre, 22 m a.s.l., *s.d., sin coll., s.n.* (CANB463451); vicinity of Mt Gillen, Alice Spring, 584 m a.s.l., *s.d., sin coll., s.n.* (CANB34861); Northern Territory Alice Spring, 582 m a.s.l., *s.d., sin coll., s.n.* (CANB221230); Todd River, 581 m a.s.l., *s.d., sin coll., s.n.* (CANB331227).

***Amaranthus crassipes* Schltdl.**

UNITED STATES OF AMERICA: Virgin Island, regularly wet plains, dumping sites, shorelines, 39 m a.s.l., *s.d., sin coll., s.n.* (HAL76208). CUBA: 16 m a.s.l., 12 October 1904, *Curitess, A. H., s.n.* (SI559). TUNISIA: Monastir, 26 m a.s.l., 10 August 2019, *sin coll., s.n.* (RO).

***Amaranthus deflexus* L.**

BOSNIA-HERZEGOVINA: Hutovo Blato, along the roads, flowerbeds, base of the walls, uncultivated terrain, 189 m a.s.l., 25 July 2007, *S. Maslo, s.n.* (RO). ROMANIA: Galati, 39 m a.s.l., 8 January 1943, *sin coll., s.n.* (RO No. 2858 d). TUNISIA: Bizerte, 5 m a.s.l., 2016, *sin coll., s.n.* (RO). ARGENTINA: Chubut, 721 m a.s.l., 30 March 1917, *sin coll., s.n.* (BA No. 32173). ITALY: Molise, Isernia, Via XXIV Maggio, 475 m a.s.l., 07 October 2021, *A.N. Hussain 3,* (IS No. D3).

***Amaranthus dubius* Mart. ex Thell.**

AUSTRALIA: Queensland, man-made habitats, 11m a.s.l., *s.d., sin coll., s.n.* (CANB589521). UNITED STATES OF AMERICA: Boquerón Colombia, 612 m a.s.l., 28 March 1935, *sin coll., s.n.* (M0312279). ZAIRE: Lwiro, 1727 m a.s.l., 28 December 1971, *sin coll., s.n.* (M0312270). MOZAMBIQUE, 11 m a.s.l., *s.d., sin coll., s.n.* (M0312273). INDIA: Poojapura, Thiruvananthapuram, 18 m a.s.l., 15 October 2021, *A. Sindhu*, 212 (UCBD15).

***Amaranthus emerginatus* Salzm. ex Uline & Bray subsp. *pseudogracilis* (Thell.) Hügin**

ITALY: Umbria, Isola Polvese, agricultural land, shorelines, artificial habitats, dumpsites, along the roads, railroads, 270 m a.s.l., 26 September 2018, *S. Bollelli & R.Penneri,* s.n. (RO); Latium, Rome, Ciampino, 115 m a.s.l., 30 July 2009, *sin coll., s.n.* (RO); Campania, Marina de Camerota, 28 m a.s.l., 15 August 2009, *sin coll., s.n.* (RO). BOSNIA-HERZEGOVINA: Mostar, Pasjak, 596 m a.s.l., 20 July 2013, *S. Maslo*, *s.n.* (RO).

***Amaranthus graecizans* L. subsp. *sylvestris* (Vill.) Brenan**

ITALY: Apulia, Bari, Along the roadsides, embankments, sandy beaches, 8 m a.s.l., 7 January 2008, *sin coll., s.n.* (RO); Umbria, Isola Polvese, 266 m a.s.l., 10 November 1991, *sin coll., s.n.* (RO); Italy: Marche, 66 m a.s.l., 16 September 2020, *sin coll., s.n.* (RO); Saudi-Arabia: Jizan, 2 m a.s.l., 17 February 2021, *sin coll., s.n.* (RO); Bosnia-Herzegovina: Mogorjelo, 10 m a.s.l., 10 July 2018, *S. Maslo, s.n.* (RO).

***Amaranthus hybridus* L.**

TURKEY: Afyon, artificial habitats, surrounding of gardens, 1007 m a.s.l., 9 November 2014, *sin coll., s.n.* (RO). SPAIN: Barcelona, 456 m a.s.l., 15 September 1920, *sin coll., s.n.* (RO No. BC972986). ARGENTINA: Berazategu, Buenos Aires, 14 m a.s.l., 21 May 1935, *Nicora E.G., s.n.* (IS No. 428). INDIA: Chitoor, Palakkad, 450 m a.s.l., 20 November 2021, *V.S.A. Kumar*, 231 (UCBD18); Italy: Umbria, Isola Polvese, 310 m a.s.l., 23 October 2007, *S. Ballelli & D. Iamonico, s.n.* (RO).

***Amaranthus hypochondriacus* L.**

Italy: Latium, Rome, Parco degli Acquedotti, man-made habitats around gardens, crop lands and along roadsides, 54 m a.s.l., 1 September 2019, *sin coll., s.n.* (RO No. 4888); Latium, Piglio abandoned rail stop station, 570 m a.s.l., 14 October 2021, *P. Fortini, s.n.* (IS); Carpinone, 648 m a.s.l., 15 August 2020, C. *Giancola, s.n.* (IS). BOSNIA-HERZEGOVINA, Banovici, 343 m a.s.l., 30 September 2020, *S. Sarie*, *s.n.* (RO).

***Amaranthus induratus* J.Palmer & Mowatt**

AUSTRALIA: near Derby western Australia, coexist in loam, red clay, along waterways, also grown in agricultural lands, 12 m a.s.l., 25 January 1971, *Allan*, *s.n.* (CANB360663); Geikie Gorge National Park, 158 m a.s.l., 17 July 1974, *G.W. Carr 3812 & A.C. Beauglehole 47590,* (CANB310085); Hall’s creek township, 416 m a.s.l., 7 December 1974, *G.W. Carr 3512 & A.C. Beauglehole 47290,* (CANB310087); Nicholson station West Australia, 197 m a.s.l., 5 July 1973, *T.E.H. Aplin 5377,* (PERTH08112371); Mount Bruce West Australia, 743 m a.s.l., 21 April 2008, *J. Alford A39,* (PERTH09141235).

***Amaranthus muricatus* (Moq.) Gillies ex Hieron.**

ITALY: Latiuo, Rome, roadsides, ruderal habitats, 26 July 2019, *D. Iamonico, s.n.* (RO No. 4878); Sicily, Palermo, Sicily, 10 January 1979, *sin coll., s.n.* (RO); Molise, Termoli, Borgo Vecchio di Termoli, Via Montecastello, 11m a.s.l., 17 July 2021, *A.N. Hussain 4,* (IS No. M4). TUNISIA: Jemmal, 33 m a.s.l., 26 July 2019, *sin coll., s.n.* (RO).

***Amaranthus palmeri* S.Watson**

ITALY: Veneto, uncultivated grounds and along the highways, 6 m a.s.l., 20 August 2018, *sin coll., s.n.* (RO No. 4845). SPAIN: Barcelona, 90 m a.s.l., 9 August 1927, *sin coll., s.n.* (RO).

***Amaranthus quitensis* Kunth.**

ARGENTINA: Formosa, Berjemo, artificial areas, man-made habitats, 162 m a.s.l., *s.d., Bayon & Moreno, s.n.* (LPAG835); La Plata, Buenos Aires, 15 m a.s.l., *s.d., Bayon N.D., s.n.* (LPAG642); La Plata, Parco Castelli Buenos Aires, 17 m a.s.l., *s.d., Bayon N.D., s.n.* (LPAG615); Plaza Belgrano, 14 m a.s.l., *s.d., sin coll., s.n.* (LPAG 633); Brizuela, Buenos aires, *s.d., sin coll., s.n.* (LPAG).

***Amaranthus rajasekharii* S.Arya, V.S.A.Kumar, W.K.Vishnu & Iamonico**

INDIA: Vidya Nagar, Kasaragod, 320 m a.s.l., 24 December 2019, *V.S.A. Kumar*, 18 (UCBD7). Poojapura, Thiruvananthapuram, 18 m a.s.l., 3 October 2020, *Arya Sindhu*, 16 (UCBD10): Kozhinjampara, Palakkad 340 m a.s.l., 6 November 2020, *Arya Sindhu*, 24 (UCBD12). Kulathupuzha, Kollam, 20 m a.s.l., 5 October 2020, *Arya Sindhu*, 17 (UCBD6). Ferry, Kochi, 30 m a.s.l., 14 August 2020, *Arya Sindhu*, 10 (UCBD3).

***Amaranthus retroflexus* L.**

ITALY: Molise, Isernia, Viale dei Pentri 76, along the roadsides, flower beds, vacant places, crop lands, railroads, 473 m a.s.l., 16 July 2021, *A.N. Hussain 5,* (IS No. R5). TUNISIA: Bizerte, 15 m a.s.l., 23 November 2015, *sin coll., s.n.* (RO). ROMANIA: Oravita, 214 m a.s.l., 1 September 2019, *sin coll., s.n.* (RO). SPAIN: Barcelona, 77 m a.s.l., 28 September 1925, *sin coll., s.n.* (RO). BOSNIA-HERZEGOVINA: Zavidovići, 216 m a.s.l., *s.d., S. Sarie, s.n.* (RO).

***Amaranthus saradhiana* Sindhu Arya, V.S.A.Kumar, W.K.Vishnu & Rajesh Kumar**

INDIA: Vithura, 300 m a.s.l., 21 August 2019, *V.S.A. Kumar*, 102 (UCBD21); Sulthan Bethery Wayanad, 550 m a.s.l., 4 September 2019, *V.S.A. Kumar*, 120 (UCBD22). Kazhakootam, Thiruvananthapuram, 10 m a.s.l., 21 August 2019, *V.S.A. Kumar*, 104 (UCBD19).

***Amaranthus spinosus* L.**

SOUTH AFRICA: Pringle Bay, shorelines, 8 m a.s.l., *s.d.*, *sin coll.*, *s.n.* (RO No. 254). UNITED STATES OF AMERICA: Texas, Port Aransas, 1 m a.s.l., *s.d., sin coll., s.n.* (RO); Italy: Liguria, *s.d., sin coll., s.n.* (RO No. n.4). ECUADOR: Guayaquil, 3 m a.s.l., *s.d., sin coll., s.n.* (RO No. 5928). INDIA: Neyyatingara, Thiruvananthapuram, 34 m a.s.l., 21 September 2022, *Arya Sindhu,* 760 (UCBD19).

***Amaranthus standleyanus* Parodi ex Covas.**

ARGENTINA: Formosa, cultivated areas, 62 m a.s.l., *s.d., Laguna Yema, N. D. Bayo’n & C. yuyo, Colorado A. Moreno*, *s.n.* (LPAG717); Salta, 1192 m a.s.l., 2 October 1951, *T.E. Luna 153*, (BA301177); Entre Rios, Concordia, 43 m a.s.l., 2 January 1949, *R. Martınez Crovetto & Leguizamo´n*, *s.n.* (SI4964). BELGIUM: Antwerp, 9 m a.s.l., 9 February 1979, *sin coll., s.n.* (TSB23483).

***Amaranthus tubercultus* (Moq) J.D.Sauer.**

ITALY: Emilia-Romagna, Ferrara, river sides, rarely in uncultivated terrain or man-made habitats, 7 m a.s.l., 20 September 2010, *sin coll., s.n.* (RO No. 4879); Friuli-Venezia Giulia, Pordenone, Sesto al Reghena, 11 m a.s.l., 30 September 2015, *sin coll., s.n.* (TSB014214); Marche, 10 m a.s.l., 23 September 2021, *N. Hoffman*, *s.n.* (IS).

***Amaranthus viridis* L.**

SAUDI ARABIA: Jizan, flower beds, along the roads, railroads, vacant places, 7 m a.s.l., 17 February 2021, *sin coll., s.n.* (RO No. n.4). TUNISIA: Bizerte, 33 m a.s.l., 23 November 2015, *sin coll., s.n.* (RO). ITALY: Molise, Isernia, via Kennedy 92, 457 m a.s.l., 29 July 2021, *A.N. Hussain 6,* (IS No. VI6). PAKISTAN: Rawalpindi, Chandni chowk, 508 m a.s.l., 14 October 2021, *A.N. Hussain 7,* (IS No. VI7). INDIA: Pangodu Thiruvananthapuram, 19 m a.s.l., 22 September 2022, *Arya Sindhu*, 710 (UCBD18).

***Amaranthus vulgatissimus* Speg.**

ARGENTINA: Buon Aires, 25 m a.s.l., 5 January 1926, *sin coll., s.n.* (BA27/2203); Salta, 1176 m a.s.l., 1 January 1943, *A. Castellanos, s.n.* (BA6708); La Rioja, 514 m a.s.l., 19 November 1927, *A. Castellanos, s.n.* (BA27/1926); San Luis, 756 m a.s.l., 27 February 1925, *A. Castellanos, s.n.* (BA No 25673); Neuquén, 340 m a.s.l., 1 January 1935, *sin coll., s.n.* (BA14361).

## References

[1] Mosyakin S.L., Robertson K.R., Flora of North America Editorial Committee (2003). *Amaranthus* L.. Flora of North America North of Mexico (Magnoliophyta: Caryophyllidae, Part 1).

[2] Hernández-Ledesma P., Berendsohn W.G., Borsch T., Von Mering S., Akhani H., Arias S., Castañeda-Noa I., Eggli U., Eriksson R., Flores-Olvera H. (2015). A taxonomic backbone for the global sunthesis of species diversity in the angiosperm orden Caryophyllales. Wildenowia.

[3] Iamonico D. (2015). Taxonomic revision of the genus *Amaranthus* (Amaranthaceae) in Italy. Phytotaxa.

[4] Iamonico D., El Mokni R. (2022). First record of *Amaranthus crassipes* subsp. *warnockii* (Amaranthaceae) out of Americas, with nomenclatural notes. Bothalia.

[5] Costea M., Sanders A., Waines G. (2001). Preliminary results towards a revision of the *Amaranthus hybridus* complex (Amaranthaceae). Sida.

[6] Das S. (2016). Amaranthus: A Promising Crop of the Future.

[7] Bayón N.D. (2015). Revisión taxonómica de las especies monoicas de *Amaranthus* (Amaranthaceae): *Amaranthus* subg. *Amaranthus* and *Amaranthus* subg. *Albersia*. Ann. Mo. Bot. Gard..

[8] Iamonico D. (2016). Nomenclature survey of the genus *Amaranthus* (Amaranthaceae). 3. Names linked to the Italian flora. Plant Biosyst..

[9] Iamonico D. (2016). Nomenclature survey of the genus *Amaranthus* (Amaranthaceae). 4. Detailed questions arising around the name *Amaranthus gracilis*. Bot. Serbica.

[10] Iamonico D. (2016). Nomenclature survey of the genus *Amaranthus* (Amaranthaceae). 5. Moquin-Tandon’s names. Phytotaxa.

[11] Iamonico D. (2020). Nomenclature survey of the genus *Amaranthus* (Amaranthaceae s.s.). 8. About *Amaranthus polygonoides s.l.* and *A. anderssonii*, two related taxa described from the tropical regions of America with notes on their taxonomy. Acta Bot. Mex..

[12] Iamonico D. (2020). A nomenclature survey of the genus *Amaranthus* (Amaranthaceae). 7. Wildenow’s names. Willdenowia.

[13] Iamonico D., Palmer J. (2020). Nomenclature survey of the genus *Amaranthus* (Amaranthaceae). 6. Names linked to the Australian flora. Aust. Syst. Bot..

[14] Mosyakin S.L., Robertson K.R. (1996). New infrageneric taxa and combinations in *Amaranthus* (Amaranthaceae). Ann. Bot. Fenn..

[15] Waselkov K.E., Boleda A.S., Olsen K.M. (2018). A phylogeny of the genus *Amaranthus* (Amaranthaceae) based on several low-copy nuclear loci and chloroplast regions. Syst. Bot..

[16] Iamonico D. (2014). *Amaranthus gangeticus* (Amaranthaceae), a name incertae sedis. Phytotaxa.

[17] Hassan N.M.S., Meve U., Liede-Schumann S. (2005). Seed coat morphology of Aizoaceae–Sesuvioideae, Gisekiaceae and Molluginaceae and its systematic significance. Bot. J. Linn. Soc..

[18] Sukhorukov A.P., Zhang M. (2013). Fruit and seed anatomy of *Chenopodium* and related genera (Chenopodioideae, Chenopodiaceae/Amaranthaceae): Implications for evolution and taxonomy. PLoS ONE.

[19] Lemus-Barrios H., Barrios D., García-Beltrán J.A., Arias S., Majure L.C. (2021). Taxonomic implications of seed morphology in *Melocactus* (Cactaceae) from Cuba. Willdenowia.

[20] Arroyo-Cosultchi G., Terrazas T., Arias S., Arreola-Nava H.J. (2006). The Systematic Significance of Seed Morphology in *Stenocereus* (Cactaceae). Taxon.

[21] Sadeghian S., Zarre S., Heubl G. (2014). Systematic implication of seed micromorphology in *Arenaria* (Caryophyllaceae) and allied genera. Flora.

[22] Amini E., Zarre S., Assadi M. (2011). Seed micro-morphology and its systematic significance in *Gypsophila* (Caryophyllaceae) and allied genera. Nord. J. Bot..

[23] Minuto L., Fior S., Roccotiello E., Casazza G. (2006). Seed morphology in *Moehringia* L. and its taxonomic significance in comparative studies within the Caryophyllaceae. Plant Syst. Evol..

[24] Hong S.P., Han M.J., Kim K.J. (1999). Systematic significance of seed coat morphology in *Silene* L. s. str. (Sileneae-Caryophyllaceae) from Korea. J. Plant Biol. Korea.

[25] Mahmoudi M., Boughalleb F., Pellegrino G., Abdellaoui R., Nasri N. (2020). Flower, seed, and fruit development in three Tunisian species of *Polygonum*: Implications for their taxonomy and evolution of distyly in Polygonaceae. PLoS ONE.

[26] Soliman M.A. (2006). Cytogenetical studies on *Aerva javanica* (Amaranthaceae). Flora Mediterr.

[27] Sindhu A., Iamonico D., Suresh V., Venugopalan Nair Saradamma A.K. (2021). First molecular and morphometric data for the genus *Allmania* (Amaranthaceae), with the description of a new species from India. Phytotaxa.

[28] Parveen M., Mitra M., Tah J., Chattopahyay N.C. (2015). Study of intraspecies variation in seed coat micro-morphology of *Amaranthus hybridus* by scanning electron microscope. Int. J. Plant Breed. Genet..

[29] Costea M., Weaver S.E., Tardif F.J. (2005). The Biology of Invasive Alien Plants in Canada. 3. *Amaranthus tuberculatus* (Moq.) Sauer var. *rudis* (Sauer) Costea & Tardif. Can. J. Plant Sci..

[30] Kamble S.R., Gaikwad D.K. (2022). Seed Morphology of Some Species of The Genus *Amaranthus* from India. Sci. Technol. Dev..

[31] Falatoury A.N., Hatami S., Torabi H., Ghezeli F., Sarani M. (2021). Taxonomic significance of inflorescence and seed characteristics in the genus *Amaranthus* in Iran. Rostaniha.

[32] Irving D.W., Beckler R. (1985). Seed Structure and Composition of Potential New Crops. Food Struct..

[33] Iamonico D. (2009). Contributo alla conoscenza del genere *Amaranthus,* L. (Amaranthaceae) nel Lazio. Proposta per una chiave analitica. Inform. Bot. Ital..

[34] Iamonico D. (2011). On the presence of *Amaranthus polygonoides* L. (Amaranthaceae) in Europe. Phyton.

[35] Iamonico D. (2017). *Amaranthus* × *romanus* (Amaranthaceae), *hybr. nov.*. Phytotaxa.

[36] Iamonico D., El Mokni R. (2017). *Amaranthus palmeri* (Amaranthaceae) in Tunisia, a second record for the continental African flora and nomenclatural notes on *A. sonoriensis nom. nov. pro A. palmeri* var. *glomeratus*. Bothalia.

[37] Iamonico D., El Mokni R. (2019). A new addition to the alien flora of Tunisia, *Amaranthus spinosus* L. (Amaranthaceae s.l.), with notes on *A. diacanthus* Raf. Acta Bot. Croat..

[38] Iamonico D., Fortini P., Noor Hussain A. (2022). On the occurrence and naturalization of *Amaranthus hypochondriacus* (Amaranthaceae) in some european countries, with notes on its climatic features. Hacquetia.

[39] Sindhu A., Iamonico D., Venugopalan Nair Saradamma A.K. (2021). *Amaranthus powellii* (Amaranthaceae), a new addition for the flora of India and a preliminary list of the indian *Amaranthus* species. Hacquetia.

[40] Thiers B. [Continuously Updated]. “Index Herbariorum: A Global Directory of Public Herbaria and Associated Staff. New York Botanical Garden’s Virtual Herbarium”. http://sweetgum.nybg.org/ih/.

[41] POWO (2023 [Continuously Updated]) *Plant of The World Online. Amaranthus* L.. https://powo.science.kew.org/taxon/urn:lsid:ipni.org:names:327362-2.

[42] Palmer J. (2009). A conspectus of the genus *Amaranthus* L. (Amaranthaceae) in Australia. Nuytsia.

[43] Iamonico D., Das S. (2014). *Amaranthus bengalense* (Amaranthaceae) a new species from India, with taxonomical notes on *A. blitum* aggregate. Phytotaxa.

[44] Cox D.R. (1972). The analysis of multivariate binary data. Journal of the Royal Statistical Society. Series C. Appl. Stat..

[45] Topliss J.G., Costello R.J. (1972). Chance correlations in structure-activity studies using multiple regression analysis. J. Med. Chem..

[46] Graham M.H. (2003). Confronting multicollinearity in ecological multiple regression. Ecology.

[47] Stewart S., Ivy M.I., Anslyn E.V. (2013). The use of principal component analysis and discriminant analysis in differential sensing routines. Chem. Soc. Rev..

[48] Aristidis L., Vlassis N., Verbeek J.J. (2003). The global k-means clustering algorithm. Pattern Recognit..

[49] Townsend C.C., Nasir E., Ali S.I. (1974). Amaranthaceae Juss. Flora of West Pakistan.

[50] Linnaeus C. (1753). Species Plantarum.

[51] Linnaeus C. (1755). Centuria I. Plantarum.

[52] Iamonico D. (2014). Lectotypification of Linnaean names in the genus *Amaranthus* L. (Amaranthaceae). Taxon.

[53] Akeroyd J.R., Tutin T.G., Burges N.A., Chater A.O., Edmondson J.R., Heywood V.H., Moore D.M., Valentine D.H., Walters S.M., Webb D.A. (1993). *Amaranthus* L.. Flora Europaea.

[54] Bolòs O., De Vigo J. (1974). Notes sobre taxonomía i nomenclatura de plantes, I. Butletí Inst. Catalana DHist. Nat. Barc. Secc. Bot..

[55] Sindhu A., Venugopalan Nair Saradamma A.K., Walsan Kalarikkal V., Iamonico D. (2020). *Amaranthus rajasekharii* (Amaranthaceae), a new species from Kerala (SW-India). Phytotaxa.

[56] Iamonico D. (2014). *Amaranthus graecizans s.l.* (*Amaranthacee*) in Italia: Note tassonomiche e distributive. Inform. Bot. Ital..

